# Aspergillus infection in chronic obstructive pulmonary diseases

**DOI:** 10.1111/crj.13585

**Published:** 2023-01-29

**Authors:** Liang Guo, Xiulin Wu, Xueling Wu

**Affiliations:** ^1^ Institute of Respiratory Disease The Second Affiliated Hospital (Xinqiao Hospital), Army Medical University Chongqing China; ^2^ Department of Geriatrics and Special Service medicine The First Affiliated Hospital (Xinan Hospital), Army Medical University Chongqing China; ^3^ Department of Respiratory Medicine Renji Hospital, Shanghai Jiaotong University School of Medicine Shanghai China

**Keywords:** chronic obstructive pulmonary disease (COPD), chronic pulmonary aspergillosis (CPA), invasive pulmonary aspergillosis (IPA)

## Abstract

Chronic obstructive pulmonary disease (COPD) is a chronic airway non‐specific inflammatory disease characterised by airway obstruction and alveolar destruction. In recent years, due to the extensive use of antibiotics, glucocorticoids, immunosuppressants and other drugs, pulmonary fungal infection in patients with AECOPD, especially aspergillus infection, has gradually increased. The forms of aspergillus infection present in COPD patients include sensitisation, chronic pulmonary aspergillosis (CPA) and invasive pulmonary aspergillosis (IPA). This review will summarise diagnostic and treatment of aspergillus in COPD patients.

## Graphical abstract

COPD is susceptible to aspergillus, and this review will summarise the diagnosis and treatment of IPA, CPA and aspergillus sensitisation in COPD patients.

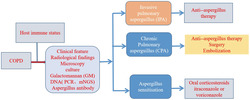

## 1 INTRODUCTION

Aspergillus is a fungus widely existing in nature, which can be spread in a large area through airborne transmission. Aspergillus spores entered into human body through the respiratory tract can cause aspergillus infection. Human respiratory system such as sinus, pharynx, tracheobronchus and lungs are the most easily affected by aspergillus. Aspergillus can be parasitic in the respiratory system, colonisation and then spread to the whole body. Traditionally, aspergillus infection was thought to occur in immunocompromised patients including severe neutropenia, haematological malignancies such as leukaemia, chronic high dose corticosteroids and haematopoietic stem cell transplantation (HSCT) and solid organ transplantation.^1^

Due to the extensive use of antibiotics, glucocorticoids, immunosuppressants and other drugs, more and more studies found that individuals with underlying chronic respiratory diseases such as COPD are susceptible to aspergillus infection.^2^ The forms of aspergillus pathogenesis in the COPD patients, including sensitisation,^3^ chronic pulmonary aspergillosis (CPA)^4^ and invasive pulmonary aspergillosis (IPA),^5^ were determined by the host immune status. Due to lacking of specific clinical manifestations and uniform diagnostic criteria, the early diagnosis of pulmonary aspergillosis in COPD patients is very difficult. This article reviews the diagnosis and treatment of aspergillus in COPD patients.

## 2 DIAGNOSTIC METHODOLOGY FOR PULMONARY ASPERGILLUS IN COPD PATIENTS

### 2.1 Culture on respiratory samples

Lower respiratory tract specimen culture is a diagnostic method for pulmonary aspergillus. However, the positive rate of aspergillus culture was different in different types of pulmonary aspergillus. The positive rate of IPA, chronic necrotising pulmonary aspergillosis (CNPA), chronic cavitary pulmonary aspergillosis (CCPA) and allergic bronchopulmonary aspergillosis (ABPA) was less than 30%. The positive rate of aspergillus nodules was even less than 10%. The culture time of aspergillus in respiratory specimens is 48–96 h.^6^, ^7^ Therefore, the low positive rate of aspergillus culture, and the long detection period limited the application in the diagnosis of pulmonary aspergillus.

### 2.2 Microscopy on respiratory samples

Compared with culture, the detection time of direct microscopy on respiratory samples is saved. However, the positive rate of aspergillus is low. The positive rate of IPA, CNPA, and CCPA was less than 10%.^8^

### 2.3 Aspergillus antigen

Aspergillus galactomannan (GM) detection is one of the classical serological methods to detect aspergillus infection, and the main detection material is galactomannan antigen. GM test in alveolar lavage fluid is more sensitive than culture and microscopy. Prior to antifungal therapy, sensitivity and specificity of GM ELISA are 80%–90% for all forms of IPA and about 75% for CPA on BALF. However, because the GM value of sputum is much higher than that of serum or BALF, the cut‐off value needs to be determined separately. However, for serum, the sensitivity of GM ELISA is 20%–80% for IPA, 20%–30% for CNPA and 10%–65% for CPA.^9^, ^10^, ^11^, ^12^ The detection period is 24–48 h.

### 2.4 Aspergillus IgG and IgM antibody

Aspergillus antibody detection is another important method for the diagnosis of pulmonary aspergillus. Detectable aspergillus immunoglobulin (Ig) G is the cornerstone of diagnosis of CPA. The sensitivity of aspergillus IgG is 70%–90%.^13^ When IgM is high and IgG is in the normal range, this indicates that the organism is recently infected with aspergillus.^14^

### 2.5 Aspergillus polymerase chain reaction

Whole blood and serum PCR can specifically amplify aspergillus DNA and further diagnose and identify aspergillus.^15^ It is not affected by antibiotic treatment and has high sensitivity and specificity, which is of great significance in the diagnosis of pulmonary aspergillosis. However, aspergillus PCR cannot distinguish between colonisation and infection.

### 2.6 Metagenomic next‐generation sequencing (mNGS)

Metagenomic next‐generation sequencing (mNGS) plays an important role in the early diagnosis of specific pathogens. Compared with the long traditional pathogen culture time and the low positive rate, the mNGS technology is fast and unbiased, which complements the shortcomings of the traditional detection methods. In recent years, mNGS have been found to play an important role in the diagnosis of pulmonary aspergillus infection. However, due to the thick cell wall of aspergillus, the detection rate of mNGS to aspergillus was low and could not distinguish between infection and colonisation.^16^

### 2.7 Lung biopsy

Pulmonary aspergillus hyphae invasion of lung tissue by pathological biopsy is the diagnostic criteria for pulmonary aspergillus. The ways to obtain lung biopsies are through fiberscopy (bronchial or transbronchial specimen) or percutaneous lung biopsy.

## 3 IPA IN COPD EXACERBATION PATIENTS

### 3.1 Risk factors

During the past decade, it was thought that the main pathogens causing acute exacerbation of COPD were viruses and bacteria. Therefore, clinical anti‐bacterial infection strategies were empirically adopted for patients with acute exacerbation of COPD. Actually, aspergillus was increasingly reported to be an important pathogen causing COPD exacerbation.^17^ The high risks of COPD suffered from IPA included four aspects: (1) systemic use prednisone more than 20 mg per day or a cumulative dose of more than 700 mg^18^; (2) high doses of inhaled glucocorticoids^19^, ^20^, ^21^; (3) COPD patients themselves^22^; and (4) viral infection including influenza and cytomegalovirus.^23^, ^24^, ^25^ Due to the high mortality of COPD patients suffered from IPA, early diagnosis and further treatment is crucial for improving survival rate for COPD patients suffering from IPA.

### 3.2 Clinical features

In contrast to haematological patients, which are characterised by fever, chest pain and haemoptysis,^26^ the clinical features of COPD patients with IPA are not atypical and mainly manifested a non‐specific antibiotic‐resistant pneumonia associated with exacerbated dyspnoea.^17^

### 3.3 Radiology

The imaging findings of IPA in COPD patients are not typical. Different from patients with haematological patients and eutropenic haematological patients, which showed wedge‐shaped consolidation, nodules, ‘halo sign’ and ‘air‐crescent sign’ imaging on thoracic CT scans, IPA imaging in COPD patients with IPA showed non‐specific consolidation and nodules.^27^, ^28^

### 3.4 Diagnosis procedure

Clinically diagnosing IPA in COPD patients remains confused. The current diagnostic criteria for IPA include EORTC/MSG, Bulpa and ICU Algorithm.^5^, ^29^, ^30^ Different populations have different diagnostic criteria for IPA due to their different host factors. The existing diagnostic criteria are graded, including proven, probable and possible. No matter which group of patients, the diagnosis criteria for proven are the same, mainly based on histopathology. Probable criteria require patients with host factors, clinical criteria and microbiological criteria. The possible criteria refers to the combination of host factors and clinical criteria, but lack of etiological evidence. Professor Bulpa's research team found that patients with chronic airway diseases such as COPD actually do not have immunosuppressive factors, although the use of glucocorticoids, probably mainly inhaled, does not use the EORTC/MSG standard, so specially proposed Bulpa standard for such patients. Probable criteria for Bulpa are as follows: (1) host factor: GOLD Grade 3 or 4 COPD patients with a history of glucocorticoids, whether inhaled, oral or intravenous use; (2) clinical criteria: the dyspnoea is not relieved by antibiotic treatment and new imaging abnormalities within the last 3 months; (3) aetiology: microscopic examination revealed aspergillus or aspergillus culture was positive or positive for specific fungal antigens or two consecutive positive blood GM tests. We can see that Bulpa criteria emphasised the risk factor that is GOLD Grade 3 or 4; actually, COPD patients with GOLD II can develop IPA in the presence of glucocorticoids.^28^ Hence, among COPD patients admitted to ICU, the Clinical algorithm seems to be more useful to diagnose IPA. Clinically, the possibility of IPA should be considered in patients with acute exacerbation of COPD treated with long‐term glucocorticoids, regardless of GOLD grade, and recent non‐specific pneumonia with exacerbation of dyspnoea that does not respond to antibiotic treatment, especially when CT shows nodules along the airway, and further examination is necessary. However, during clinical practice, diagnosis of IPA in COPD patients is difficult. The limitations include the following aspects: (1) The clinical features are not typical. Compared with haematological patients with IPA, the classic symptoms such as fever, cough, chest pain and haemoptysis are less common in COPD patients with IPA, mainly manifested as non‐specific antibiotic‐resistant pneumonia associated with exacerbated dyspnoea.^17^, ^31^ (2) COPD acute exacerbation patients are less likely to undergo bronchoscopy or percutaneous lung biopsy to obtain histological specimens; therefore, it is difficult to ascertain IPA in COPD patients. (3) Radiology of COPD patients with usually presents as infiltrates or consolidations or nodules, lacking of typical imaging of IPA, such as ‘halo sign’ or ‘air crescent’ that were usually seen in neutropenic patients with IPA.^27^, ^28^ (4) Microbiological and culture confirmation in COPD patients with IPA is limited. Firstly, it is difficult to obtain valid lower respiratory tract specimens for patients with COPD III‐IV. Secondly, the sensitivity of aspergillus in lower respiratory samples is low. Thirdly, it is difficult to distinguish between colonisation and infection even if aspergillus was detected in the LRT.^32^, ^33^ (5) Although BALF galactomannan (GM) is the most common diagnostic test used in clinical practice, difficult‐to ‐two consecutive BALF species limited the use of GM for diagnosing IPA in COPD patients. In addition, when patients were treated with antibiotics, especially piperacillin–tazobactam and amoxicillin–clavulanic acid, the GM results will be false positives.^34^, ^35^ (6) Due to glucocorticoid use and immune state in COPD patients, antibody detection is not suitable for IPA in COPD patients and more likely for diagnosing CPA.^32^ (7) PCR requires high technical difficulty and cannot distinguish between colonisation and infection. (8) Due to the thick cell wall of aspergillus, the detection rate of mNGS to aspergillus was low and could not distinguish between infection and colonisation; therefore, it is not recommended to use alone. Therefore, for COPD patients with high risk of IPA, combination of multiple assays is necessary for the diagnosis of IPA in COPD patients.

### 3.5 Treatment

**FIGURE 1:**
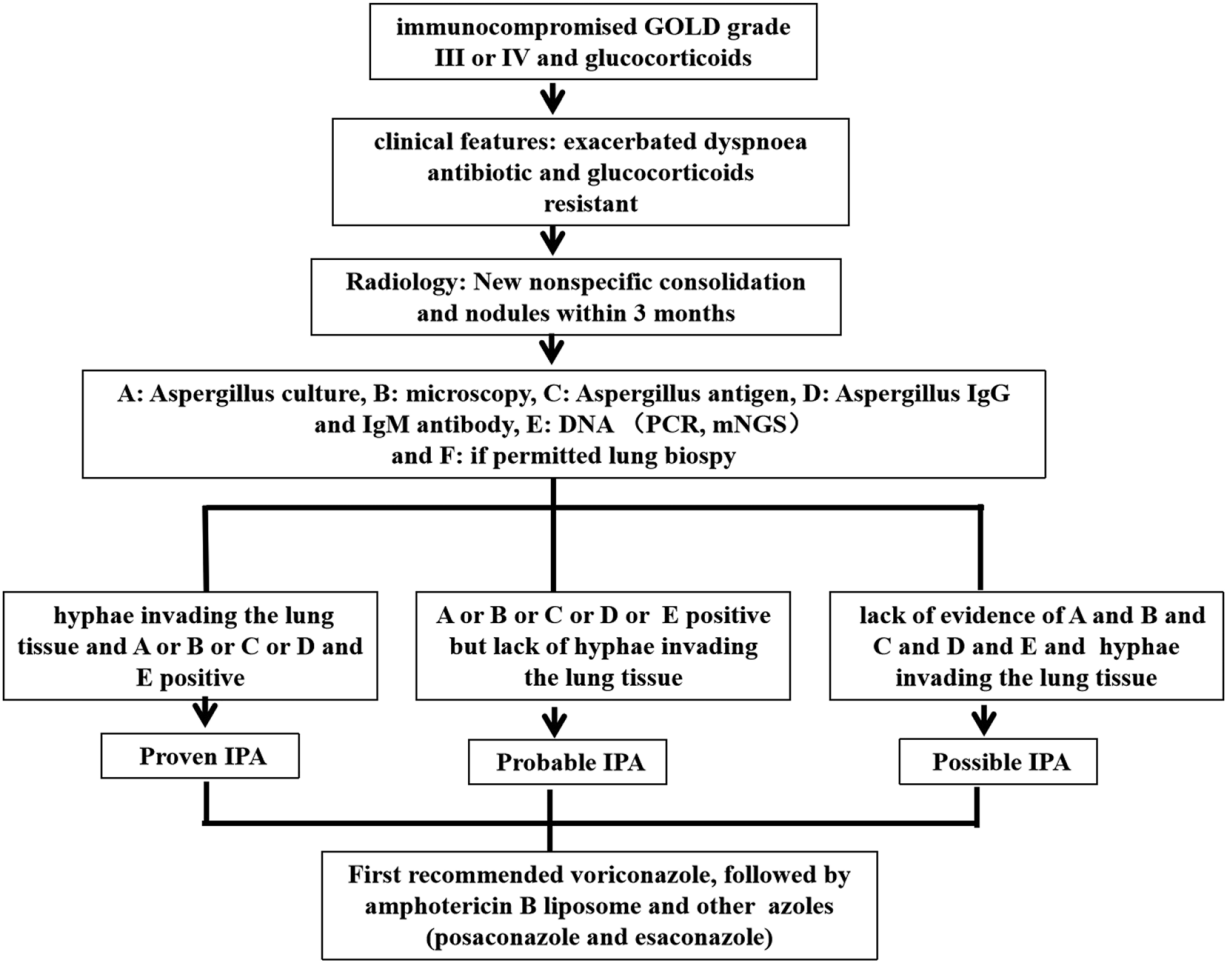
Modified diagnostic procedure and treatment for IPA in COPD patients

Prophylactic anti‐aspergillus therapy is generally not suggested in patients with chronic obstructive pulmonary disease.^36^ Antifungal therapy is recommended for patients with proven and probable patients.^37^ For possible, empiric anti‐therapy may be considered in patients that are with high risk factors, recent exacerbation of dyspnoea, failure to respond to antibiotic therapy and/or failure to glucocorticoids and recently increased nodule distribution along the airway. Voriconazole is recommended as the first choice of treatment,^38^ followed by amphotericin B liposome, and other azoles, such as posaconazole and esaconazole,^39^, ^40^ are less frequently used due to fewer reports in the literature. The diagnosis procedure and treatment were seen in Figure 1.

## 4 CPA IN COPD PATIENTS

### 4.1 Risk factors and classification

CPA, most commonly caused by *Aspergillus fumigatus*, is a slowly progressive pulmonary disease, which manifested as significant respiratory and systemic symptoms associated with significant morbidity and mortality.^41^, ^42^ The occurrence of CPA is closely related to the host immune state and tends to occur in patients with underlying lung diseases, such as history of tuberculosis,^43^, ^44^ active atypical mycobacterial infection,^45^, ^46^ COPD,^47^, ^48^ asthma,^49^ treated lung cancer^41^ and sarcoidosis.^50^ CPA has five subgroups: simple aspergilloma, CCPA, CNPA, aspergillus nodule and chronic fibrosing pulmonary aspergillosis (CFPA).

### 4.2 Clinical features

CPA tends to occur in middle‐aged men with systemic symptoms such as weight loss, general fatigue, night sweats and decreased appetite, chronic cough, dyspnoea, chest discomfort and occasional haemoptysis. However, the clinical symptoms of different types of CPA are different.

### 4.3 Simple aspergilloma

The clinical manifestations of pulmonary aspergillus globules are not specific and not restricted by age and sex. The most prominent clinical symptom is repeated intermittent haemoptysis. Most aspergilloma patients mainly have a small amount of haemoptysis, and a few can have fatal massive haemoptysis, which was most commonly seen in patients with tuberculous aspergilloma.^51^

### 4.4 CCPA

CCPA patients mainly present with subacute symptoms such as cough, chest pain and a small amount of haemoptysis, but some patients present with symptoms similar to those of tuberculosis, such as fever, chills, night sweats and weight loss.^52^

### 4.5 CFPA

Extensive pulmonary fibrosis involving at least two lobes with CCPA results in patients' severe impairment of lung function. In addition to the clinical manifestations similar to CCPA, there is shortness of breath or dyspnoea, especially after exercise.^53^

### 4.6 Aspergillus nodule

Most aspergillus nodule patients are asymptomatic and are usually detected by chest imaging.

### 4.7 CNPA

CNPA is also known as subacute invasive pulmonary aspergillosis, which usually has invasive features, characterised by chronic cough, expectoration, fever and systemic symptoms. Fifteen per cent of CNPA patients had haemoptysis.^54^

### 4.8 Radiology

Imaging of CPA includes two aspects: the manifestation of CPA itself and the manifestation of the underlying lung disease associated with CPA. The typical imaging appearance of aspergilloma is ‘air‐crescent sign’, that is, a solid, round or oval luminal mass was partially surrounded by a crescent of air.^55^ The typical imaging of CCPA is unilateral or bilateral consolidation and multiple progressive thick‐walled cavities that may contain one or more aspergillus pellets with varying degrees of pleural thickening.^56^

### 4.9 Diagnosis

CPA diagnosis is required to satisfy the following conditions^13^, ^57^: (1) typical chest imaging findings; (2) direct evidence of aspergillus infection or a positive immune response against aspergillus; (3) The course of disease is at least 3 months and other diseases are ruled out. Therefore, in patients with underlying pulmonary disease, chest imaging should be performed once clinical manifestations of CPA are present. If chest imaging indicates aspergilloma, aspergillus IgG or precipitate tests should be performed, and aspergillus infection can be confirmed in patients with >90% positive results. If the antibody test is negative, evidence of additional aspergillus infection, such as aspergillus microscopy, culture, GM and mNGS even lung biopsy, is required. If chest imaging indicates one or more cavities, aspergillus microscopy, culture, GM, PCR or mNGS even lung biopsy needs to perform. When these results are positive, pulmonary aspergillosis is diagnosed. SAIA was diagnosed in immunocompromised or highly depleted patients who met the diagnostic criteria for IPA but progressed more slowly than acute invasive aspergillosis, with a disease course of 1–3 months.

### 4.10 Treatment

**FIGURE 2:**
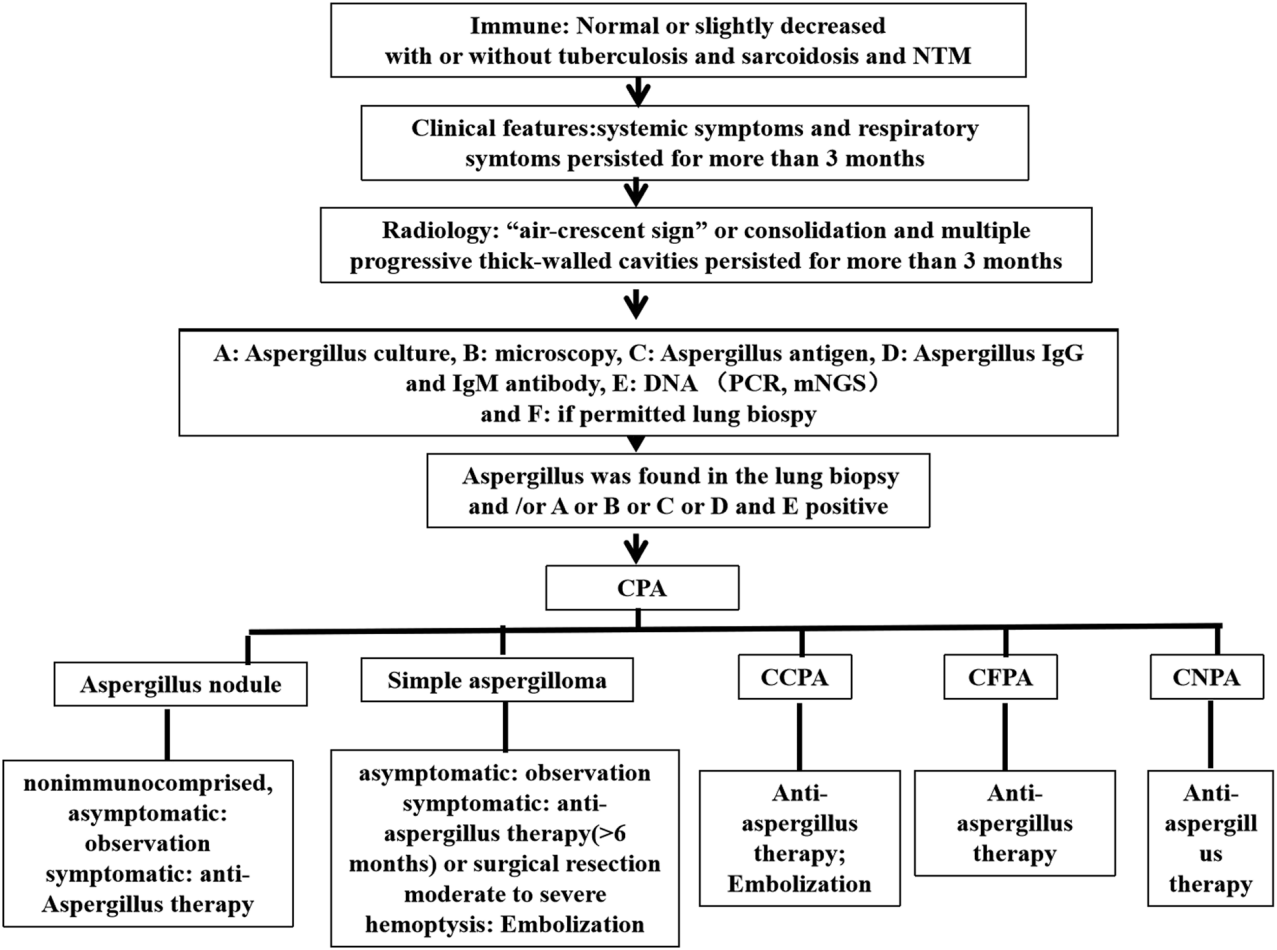
Diagnostic procedures and treatment for CPA in COPD patients

In clinical practice, antifungal therapy should be decided based on the types of CPA patients, clinical manifestations and surgical indications. The aim of antifungal therapy is to control infection, prevent the progressive development of pulmonary fibrosis, prevent haemoptysis and improve the quality of life. Current guidelines and studies recommend antifungal therapy for symptomatic patients. Oral antifungal therapy is recommended for at least 6 months in symptomatic patients or CPA patients with progressive disease. The use of triazoles is recommended in the latest guidelines for CPA issued by European Federation of Clinical Microbiology and Infectious Diseases (ESCMID), European Respiratory Society (ERS).^36^ The diagnosis procedure and treatment were seen in Figure 2.

### 4.11 Simple aspergilloma

For patients with simple aspergilloma with normal immune function, stable and asymptomatic, no changes in imaging after 3 months of continuous observation usually do not require antifungal therapy, and continuous observation is recommended.^38^ For unstable patients, surgical resection was performed on the basis of antifungal therapy (generally more than 2–4 weeks), and antifungal therapy was performed for 3 months after resection. For those who are contraindicated for surgery, local infusion of drugs is recommended. For mild haemoptysis, guidelines recommend tranexamic acid for treatment. Embolisation is necessary for patients with moderate to severe haemoptysis.^57^, ^58^

### 4.12 CCPA

Oral azoles are considered the standard of care for CCPA. For patients with limited CCPA lesions, if long‐term antifungal therapy is ineffective or cannot be tolerated, surgical treatment can be attempted. Generally, 2 weeks of antifungal therapy before surgery is recommended, followed by a moderate or long course of continuous therapy after surgery, depending on the immune status of the patient. In patients with severe haemoptysis, bronchial artery embolisation should be performed.^57^, ^58^

### 4.13 CFPA

For CFPA, the target is slowing down the progression of lung function and improving dyspnoea symptom, suggesting long‐term treatment.^59^

### 4.14 Aspergillus nodule

For a single aspergillus nodule, non‐immunocompromised, asymptomatic patient, suggesting continued observation. For symptomatic patients, anti‐aspergillus therapy is recommended.^60^

### 4.15 CNPA

For CNPA, the treatment strategy is the same as IPA.^61^

### 4.16 Aspergillus sensitisation in COPD patients

Numerous studies demonstrated that aspergillus sensitisation was significantly associated with poor clinical outcomes in asthma, bronchiectasis and cystic fibrosis.^44^, ^45^, ^46^ Also, aspergillus sensitisation was found to occur in patients with COPD.^62^ Aspergillus sensitisation is confirmed by aspergillus IgE, which was detected in serum or on positive skin prick.^63^ COPD, which was one of the most common groups to manifest aspergillus sensitisation, accounted for 35%–55%. In most cases, aspergillus sensitisation was considered to highly associate with greater symptoms, more frequent exacerbations and poorer lung function. But other part of studies reported aspergillus sensitisation had no association with COPD exacerbations and poorer lung function.^64^, ^65^ Meanwhile, radiology was not related with aspergillus sensitisation, and aspergillus or aspergillus IgG positivity was not linked to airway or lung infection.

### 4.17 Treatment

**FIGURE 3:**
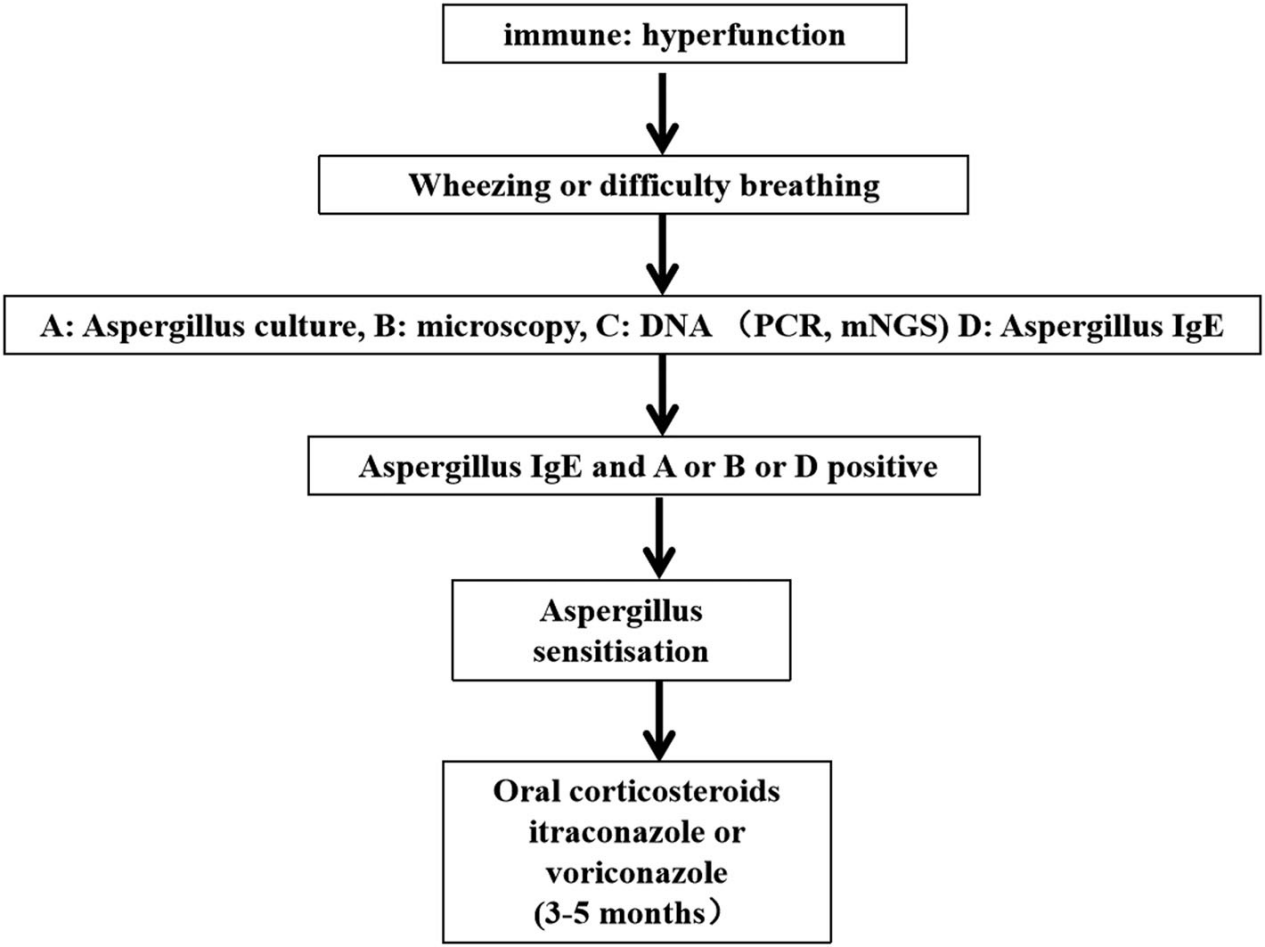
Diagnostic procedures and treatment for aspergillus sensitisation in COPD patients

Oral corticosteroids are the main treatment for aspergillus sensitisation. Treatment with corticosteroids can increase the resolution of radiographic infiltrates and reduce serum total IgE and peripheral eosinophilia. To improve symptoms, facilitate weaning from corticosteroids, decrease aspergillus titres and improve radiographic abnormalities and pulmonary function, itraconazole or voriconazole has been suggested in aspergillus sensitisation of COPD patients. The treatment course is 3–5 months.^66^, ^67^ The diagnosis procedure and treatment were seen in Figure 3.

## 5 CONCLUSION

Aspergillus infection exists in COPD patients, especially in Grade 3–4 COPD patients, and its existence forms are related to host immune, including IPV, CPV and aspergillus sensitisation. For COPD patients with IPV, the clinical manifestations are not typical. For COPD patients with high risk factors for IPA, the possibility of IPA should be considered, and a combination of multiple appropriate methods should be used for diagnosis as early as possible. Voriconazole is recommended as a first‐line agent for the proven, probable and possible treatment of IPA. However, prophylactic anti‐aspergillus therapy is generally not given in patients with COPD. For COPD patients with CPV, different types of CPA have different clinical manifestations, imaging, preferred examination methods and treatment. Treatments include anti‐aspergillus therapy, surgery, haemostasis and interventional embolisation. For aspergillus sensitisation in COPD, specific aspergillus IgE as well as skin prick tests are helpful in confirming the diagnosis. Glucocorticoid, itraconazole and voriconazole are the main drugs for the treatment of aspergillus sensitisation in COPD.

## AUTHOR CONTRIBUTIONS

Liang Guo drafted the article; Xiulin Wu searched the literature and modified the paper; Xueling Wu conceived, designed and supervised the study.

## CONFLICT OF INTEREST

The authors report no declarations of interest.

## Data Availability

Data sharing is not applicable to this article as no new data were created or analyzed in this study.
